# Evidence from single nucleotide polymorphism analyses of ADVANCE study demonstrates *EFNB3* as a hypertension risk gene

**DOI:** 10.1038/srep44114

**Published:** 2017-03-08

**Authors:** Johanne Tremblay, Yujia Wang, John Raelson, Francois-Christophe Marois-Blanchet, Zenghui Wu, Hongyu Luo, Edward Bradley, John Chalmers, Mark Woodward, Stephen Harrap, Pavel Hamet, Jiangping Wu

**Affiliations:** 1 Research Centre, Centre hospitalier de l’Université de Montréal (CRCHUM), Montreal, Quebec H2X 0A9, Canada; 2 The George Institute for Global Health, University of Sydney Sydney, New South Wales, 2006, Australia; 3 The George Institute for Global Health, University of Oxford, Oxford, UK; 4 Department of Epidemiology, Johns Hopkins University, Baltimore MD, USA; 5 Department of Physiology, University of Melbourne, Victoria 3010, Australia

## Abstract

EPH kinases and their ligands, ephrins (EFNs), have vital and diverse biological functions. We recently reported that *Efnb3* gene deletion results in hypertension in female but not male mice. These data suggest that EFNB3 regulates blood pressure in a sex- and sex hormone-dependent way. In the present study, we conducted a human genetic study to assess the association of *EFNB3* single nucleotide polymorphisms with human hypertension risks, using 3,448 patients with type 2 diabetes from the *ADVANCE* study (Action in Diabetes and Vascular Disease: Peterax and Diamicron MR Controlled Evaluation). We have observed significant association between 2 SNPs in the 3′ untranslated region or within the adjacent region just 3′ of the *EFNB3* gene with hypertension, corroborating our findings from the mouse model. Thus, our investigation has shown that *EFNB3* is a hypertension risk gene in certain individuals.

EPH erythropoietin-producing human hepatocellular carcinoma receptor kinases are the largest family of receptor tyrosine kinases. They are divided into A and B subfamilies according to sequence homology^1^. Ephrins (EFNs), which are also cell surface molecules, are ligands of EPHs. EFNs are classified into A and B subfamilies. Members of the A subfamily attach to the cell surface through glycosylphosphatidylinositol anchoring, whereas members of the B subfamily attach through transmembrane tails^2^,^3^,^4^. Interactions among EPHs and EFNs are promiscuous but, in general, EPH A members interface preferentially with EFN A family members, and EPH B members with EFN B family members^2^,^3^,^4^. Such redundancy suggests that these kinases are crucial in various biological contexts. EFNs can stimulate EPH receptors, and this is called forward signaling. Interestingly, EPHs are also capable of stimulating EFNs which then transmit signaling reversely into cells, a phenomenon known as reverse signaling.

EPHs and EFNs are expressed in many tissues and organs. They play important roles in the central nervous system^2^,^4^, immune system^5^,^6^,^7^,^8^,^9^,^10^,^11^,^12^,^13^,^14^, digestive system^15^, bone metabolism^16^,^17^, angiogenesis^18^ and other processes^19^,^20^,^21^,^22^,^23^,^24^,^25^.

We recently reported that members in the EPH/EFN families*, i.e.,* EPHB6, EFNB1, EFNB3, EFNB2 and EPHB4 are crucial in blood pressure (BP) regulation in mouse models^26^,^27^,^28^,^29^,^30^,^31^. While the default function of the first 3 is to reduce BP, the default function of the last 2 is opposite, *i.e.,* to increase BP: gene deletion of *Ephb6, Efnb1* and *Efnb3* results in increased BP, but the deletion of *Efnb2* and *Ephb4*, decreased BP. These genes are expressed in vascular smooth muscle cells (VSMCs), providing a molecular framework for their function in these cells. Through smooth muscle-specific gene deletion and *in vitro* mechanistic dissection, we have proved that VSMCs are the major targets through which EPHs/EFNs exert their effect on BP modulation^26^,^27^,^28^,^29^,^30^,^31^. The BP phenotype in the knock out (KO) mice is often sex- and sex hormone-level dependent. For example, hypertension in EPHB6 KO is only observed in castrated mice^26^; hypertension after *Efnb3* KO is present in females but not males; the BP phenotype in *Efnb3* KO mice is reversed after gonadectomy: castrated KO males become hypertensive, while ovariectomized KO females, normotensive^29^; on the other hand, male but not female *Efnb2* and *Ephb4* KO mice are hypotensive^27^,^30^.

The findings from the above-mentioned mouse studies prompted us to investigate the relevance of EPHB and EFNB BP-regulating function to human hypertension. Indeed, we have previously revealed that 5 single nucleotide polymorphisms (SNPs) in the *EFNB2* gene are associated with hypertension in male patients in the *ADVANCE* (Action in Diabetes and Vascular Disease: Peterax and Diamicron MR Controlled Evaluation) study^30^, a clinical trial in type 2 diabetes (T2D)^32^. Also, a SNP in the *GRIP1* gene, whose product is in the EFNB3 signalling pathway, is approaching the *Boferroni*-corrected *p*-value in International Blood Pressure Consortium cohorts^31^.

As a continued effort to establish the relevance of our mouse findings to human hypertension, we queried the association of SNPs in the EFNB3 gene to hypertension in *ADVANCE*. We restricted our query specifically to EFNB3, excluding other EPH/EFN genes to a penalty from excessive multiple testing. The results reveal that 2 SNPs in the *EFNB3* gene were significant for their association with hypertension in T2D patients.

## Methods

### Patient population

The patient cohort consisted of 3,448 patients with T2D of European descent and at high risk for macrovascular or microvascular diabetes complications who were recruited in the *ADVANCE* clinical study, a factorial, multicentre, randomised controlled clinical trial of 11,140 participants recruited from 215 centers in 20 countries^32^,^33^. All individuals were T2D subjects 65 years old or older, or they were T2D subjects 55 years old or older who were diagnosed at age of 30 years or older, and had one of the following: a history of major macrovascular disease; a history of major microvascular disease; diagnosis of T2D over 10 years prior to entry into study; presence of another major risk factor for vascular disease including: smoking, dyslipidemia, microalbuminuria.

The 3,448 patients were classified as normotensive or hypertensive. Hypertension was defined as treated for hypertension or having a measurement of systolic pressure >140 mm Hg or diastolic pressure >90 mmHg at entry into the study. As detailed in Table 1, 70.5% and 78.8% of male and female cases, respectively, who were admitted into the study were under anti-hypertensive treatment, This 140/90 mmHg threshold for hypertension diagnosis for the remaining cases and controls was adopted according to the U.S. National Institute of Health guidelines proposed for hypertension diagnosis^34^, and this threshold is used in most clinical trials including the *ADVANCE* study^32^.

There were 2219 males in the cohort of whom 1794 were hypertensive and 425 were normotensive. There were 1229 females of whom 1016 were hypertensive and 213 were normotensive. There were 4.4 times as many hypertensive as normotensive diabetic subjects and 1.8 times as many males as females.

### Ethics statement

This human genetic study was carried out in accordance with relevant guidelines of the participating institutions. The research protocol was approved by ADVANCE Study Ethics Committees. Informed consent was obtained from all the subjects used in this study.

### Genotyping and data analysis

The 3,448 individuals were genotyped for 724,847 SNPs with Affymetrix Genome-Wide Human SNP Arrays 5.0 or 6.0 at the genomic platform of the CRCHUM. A further 8,117,344 SNP genotypes were then imputed with the IMPUTE2 program^35^,^36^. A subset (147,088) of the genotyped SNPs that were in Linkage equilibrium (at *r*^*2*^ ≤ 0.8) was selected to perform a principal component (PC) analysis. using the Eigenstrat software^37^ in order to test for population stratification in the European wide sample. The first two independent principal components from this analysis (PC1 and PC2) which account for the majority of the covariance among genotypes due to population structure were able to clearly separate samples according to geographic origin within Europe and were subsequently used as covariates in the association analysis to correct for any population stratification effects. Association analysis was performed for 39 SNPs, 2 genotyped and 37 imputed, that fell within the 25,066-bp region of the *EFNB3* gene plus additional 10-kb stretches 5′ and 3′ of the gene, between positions 7,599,260 and 7,624,326 (Build 37/hg19) on chromosome 17. Association analysis was performed for males-only, females-only and combined male and female samples of hypertensive patients versus normotensive controls using SNPTEST, the companion program to IMPUTE2^35^,^36^. A logistic regression model with additive genotype coding and with PC1, PC2, age, body mass index (BMI), and genotyping batch and genotyping array (5.0 Vs. 6.0) as covariates was used to perform the association tests. Sex was also included as a covariate for the combined analyses of males and females. The number of tagging SNPs among the 39 analyzed SNPs located within the gene region was determined for an *r*^2^ value > 0.8 and a minor allele frequency cut-off of 0.05 using the LDSELECT program^38^. Seven tag SNPs were identified across the *EFNB3* region for the CEU population (Utah residents with ancestry from northern and western Europe), indicating the presence of 7 independent LD blocks and 7 independent tests across these genotypes. We therefore corrected for 7 independent tests for 3 sets of analysis (males only, females only and combined) resulting in 21 independent tests and thus used a *Bonferroni* significance threshold of 0.05/21 giving a critical *p*-value of 0.0024, or a critical −log10 *p*-value of 2.62. We note that this correction is conservative. The male only and female only samples are subsets of the combined sample, so that the combined sample does not represent a set of completely new independent tests. Nevertheless the patterns of association of SNPs are such that this over-correction is of little concern (see Locus Zoom plots below).

## Results

**Figure 1:**
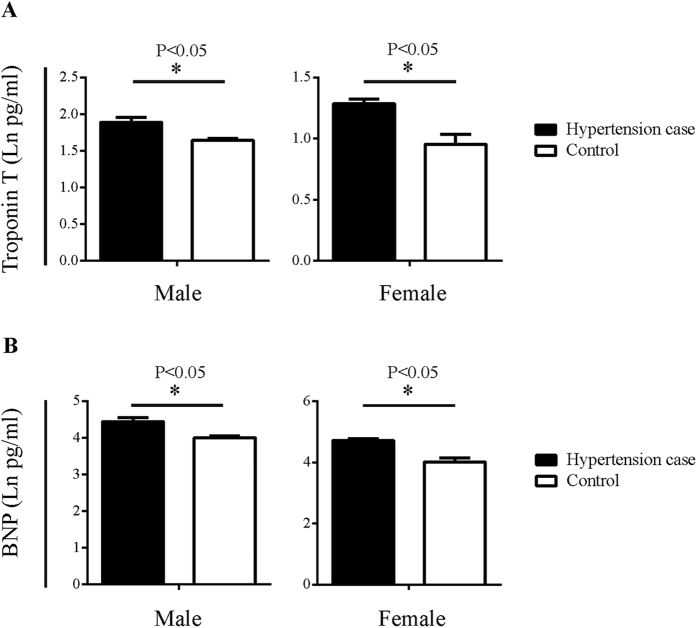
Serum troponin T and BNP levels in hypertension case subjects and controls. The data were converted to natural log (Ln) to achieve normal distribution for Student’s *t* test. Means + SD are presented. Sample sizes (n) are indicated. *p < 0.05. (**A**) Serum troponin T levels in male (left panel) and female (right panel) subjects (**B**) Serum BNP levels in male (left panel) and female (right panel) subjects.

The levels of two cardiovascular disease-related biomarkers, troponin T and brain natriuretic peptide (BNP), in the sera of the hypertension case and control subjects were registered. The levels of these two biomarkers were significantly elevated in both male and female cases, compared to those of the controls (Fig. 1). High serum troponin T levels reflect myocardium damage^39^, which is often a complication of hypertension, while BNP levels have been reported to be progressively elevated with increasing severity of hypertension, particularly when left ventricular hypertrophy is present^40^. Thus, the elevation of both these biomarkers in the hypertension cases serves as a validation for the presence of hypertension and/or its complications in these subjects.

**Figure 2:**
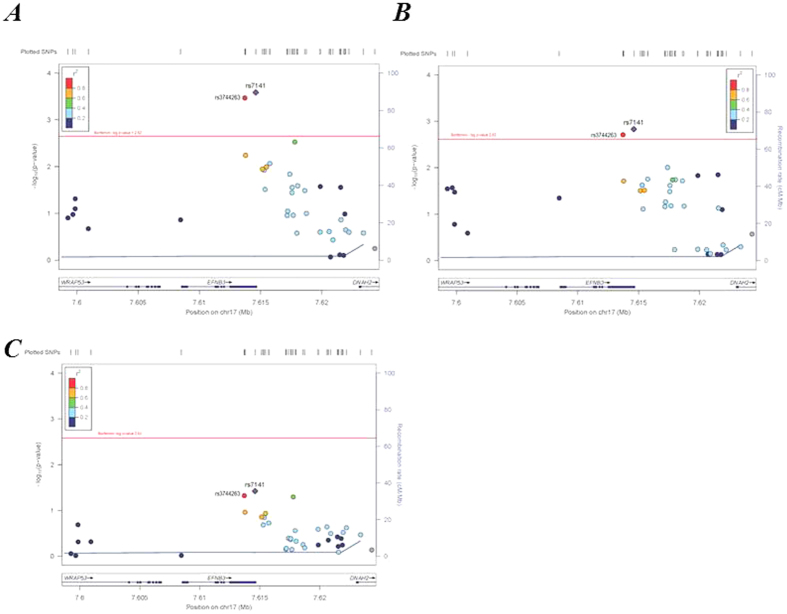
LocusZoom plots of –log_10_ p-values and r^2^ values of SNPs for the association, and rates of recombination in the regions analyzed according to 5 genes examined in the ADVANCE study. The symbols (circles and diamonds) and the left-hand vertical axis illustrate –log_10_ *p-value*s of the SNPs. Blue lines and the right-hand vertical axis represent rates of recombination in the chromosomal regions concerned. Red lines represent the Bonforroni-corrected *p*-value. The color of the symbols represents *r*^2^ values. The horizontal axis indicates the position in the chromosomes. The position of the *EFNB3* gene is shown in the bottom of the figures.

**Figure 3:**
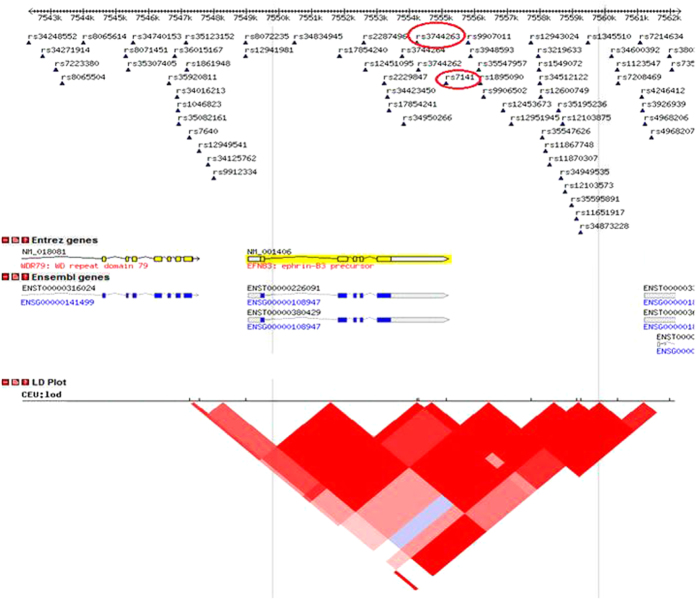
Location of associated SNPs with respect to LD structure in 3′ region of EFNB3 The red ovals indicate the position of the 2 SNPs in the *EFNB3* gene, which have significant association with hypertension in T2D patients from the *ADVANCE* study. The red-color triangle is a plot of LD strength between the 2 said SNPs.

The results of association analysis of the 39 SNPs present within the *EFNB3* gene and surrounding regions and hypertension are presented in Table 2 and Fig. 2. Two SNPs with reference SNP cluster ID 3744263 (*rs3744263)* (genotyped) and *rs7141* (imputed) located within the 3′ untranslated region (3′UTR) of the *EFNB3* gene were associated with hypertension at *p*-values below the *Bonferroni* critical *p*-value for the combined sample (0.00034 and 0.00026, respectively) and for the males-only sample (0.00148 and 0.00195, respectively). The *p*-values for the females-only sample for these SNPs were nominally significant but not significant when corrected for multiple testing (0.047 and 0.038, respectively). These two SNPs were in high linkage disequilibrium (LD) (*r*^*2*^ = 0.896, CEU) (Fig. 3), so that most likely they are both serving as proxy for a functional polymorphism, probably located within the 3′UTR of *EFNB3*.

Table 3 shows the allele frequencies for the two SNPs, *rs7141* (A and G) and *rs3744263* (C and T) along with odds ratios and their confidence limits for the combined sample, and for the male-only and the female-only samples. For both SNPs, the major allele (A or C) was more frequent among cases than among controls, in combined sample, males-only sample and females-only sample. The minor allele (G or T) was more frequent among controls in all the samples. Both SNPs had essentially the same major allele odds ratios in all the samples, which were higher among males than females (1.364 versus 1.249 for *rs7141*, 1.364 versus 1.242 for *rs3744263*), although the difference was not significant.

Table 4 presents the results of a test of independence of allele frequencies between males and females for cases and controls. Although the associations between hypertension and the 2 SNPs were significant in males and combined samples but not in females as shown above in Table 1, the *χ*^*2*^ tests of independence in both sexes were not significant for either cases or controls, suggesting that allele frequencies are not significantly different between cases and controls in both sexes. The major allele frequencies for both SNPs (A and C) were slightly higher among the male cases, but the difference was not significant. The major allele frequencies among male and female controls were essentially the same. These tests suggest that the association of the 2 SNPs to hypertension is similar between male and female subjects.

## Discussion

Our genetic investigation in patients recruited from the *ADVANCE* study revealed significant association of 2 SNPs within the *EFNB3* gene with hypertension in T2D patients. This finding corroborates our results obtained from *Efnb3* KO mice based on *in vivo, ex vivo* and *in vitro* experiments, which showed that *EFNB3* deletion results in hypertension^29^.

In this human genetic study, we have two inclusion criteria for hypertension cases: 1) patients under active anti-hypertensive treatment; the majority (73.5%) of the cases are in this category; or 2) patients with >140 mmHg for systolic or >90 mmHg for diastolic pressure. The 140/90 mmHg threshold is adopted for this study based on the U.S. National Institute of Health guidelines^34^, and this threshold is also used in most clinical trials including the *ADVANCE* study^32^. There could exist some “white-coat hypertension” when the 140/90 mmHg threshold is used. However, the reported incidence of “white-coat hypertension” is about 15–30% in subjects diagnosed with elevated office blood pressure measurements^41^,^42^. In our study, since only 26.5% of the hypertension cases were recruited based on office blood pressure measurement at the time of recruitment, the possible contamination of “white-coat hypertension” in this group will only be 15–30% of 26.5%, *i.e*., a negligible 4–8% of the total hypertension cases.

If we adopt a higher BP cut-off threshold for the inclusion criteria, such as >160/100 mmHg, to reduce the potential contamination from “white-coat hypertension”, we will not be able to use all those patients recruited based on prior diagnosis and under active anti-hypertensive treatment as cases. Therefore, we would lose 70.5% and 78.8% of our total male and female cases, respectively, who are certainly not experiencing “white-coat hypertension”. This will greatly reduce our statistical power. Based on these considerations, for better statistical power, we adopted the classical 140/90 mmHg threshold for the definition of hypertension for those recruited based on their BP measurements.

No genes in the *EPHB/EFNB* family were identified as hypertension risk genes in several large-scale GWAS^43^,^44^,^45^,^46^,^47^,^48^,^49^,^50^,^51^, including that of the International Blood Pressure Consortium (IBPC)^43^. It is possible that the contribution of genes in the EPHB/EFNB family to the BP phenotype is relatively small, and the possible association is rendered undetectable due to heavy multiple testing statistical penalties in GWAS. We then specifically queried the IBPC dataset for association of *EFNB3* and a group of related genes with blood pressure. The results have been published recently^31^. The SNPs in the region of *EFNB3* gene are highly under-represented in the ICBP meta-analysis genotype map, and no significant association of available *EFNB3* SNPs with blood pressure is revealed. However, we noticed that the *p*-value of a SNP in *GRIP1*, a signalling molecule in the EFNB3 signalling pathway, is 0.000389, approaching the *Bonferroni*-corrected critical *p*-value of 0.000302. Considering the very conservative nature of *Bonferroni* correction, it is indicative of the implication of EFNB3 in BP control.

Why can we detect the association of 2 *EFNB3* SNPs with hypertension in the *ADVANCE* study but none in the IBPC dataset? One possible reason is that these 2 studies have different inclusion criteria of samples. The ICPB meta-analysis avoids diabetic patients and attempts to reflect the range of blood pressures of the general population, while the *ADVANCE* samples are all diabetic patients. Then why can we detect a significant *GRIP1* SNP but not *EFNB3* SNPs in the IBPC study? GRIP1 is in the signalling pathways of several other EFN/EPH molecules (*e.g*., EPHB6 and EFNB1), and these molecules are also involved in BP regulation^26^,^28^; consequently, *GRIP1* might carry higher statistical weight than *EFNB3* in BP control, hence the detection of its SNP association with BP.

The 2 SNPs for which significant association was found (Table 2) are all in the 3′ untranslated region or within the adjacent region just 3′ of the *EFNB3* gene (Fig. 2), and are in LD (Fig. 3). None of the 2 SNPs were found to alter directly sequences involved in well-defined functions such as messenger stability (UA-rich sequences), binding sites for microRNA species which may induce increased mRNA turnover, or alter the poly (A) tail which is also involved in mRNA stability. These SNPs are in LD and may be serving as proxy for an unidentified polymorphism that is affecting these functions. However we are not able, at this time, to pin-point the exact functional polymorphism.

In mice, the female *Efnb3* KO manifested BP increase, but in female T2D patients, the 2 SNPs in the *EFNB3* gene were not significantly associated with hypertension. There was no significant difference between males and females, for both cases and controls, with regard to major allele frequency. A lack of detection of the association of 2 SNPs to hypertension in female was possibly the result of insufficient statistical power due to a small female T2D patient sample size, as only 1229 females (1016 hypertensive and 213 normotensive) were genotyped. A second reason could be that the T2D females in the ADVANCE study are all above 55 year old, and likely to have reduced estrogen levels. We demonstrated that EFNB3′s role in BP was estrogen-dependent, as female KO mice after ovariectomy no longer had increased BP^29^. It is therefore conceivable that with a larger female patient sample size and a younger population, these 2 SNPs might become significantly associated with hypertension in females.

In mice, *Efnb3* KO males were normotensive and they only became hypertensive after castration, indicating that testosterone in the absence of EFNB3 is protective against hypertension. However, in male T2D patients, 2 *EFNB3* SNPs (*rs7141* and *rs3744263*) were significantly and positively associated with hypertension. There could be several possible explanations. 1) The minimal age of male patients in the ADVANCE study was 55-years old. Plasma total testosterone levels range from 270 to 1,070 ng/dL in “normal” adult males^52^. Considering 346 ng/dL as a cut-off for the diagnosis of hypogonadism, as recommended by the International Society for the Study of the Aging Male, about 30% of men older than 40 years are hypogonadic^53^. Further, male patients with the metabolic syndrome are prone to hypogonadism^54^. Therefore, it is conceivable that more than 30% of the male patients in the *ADVANCE* study suffer from hypogonadism, reminiscent of the reduced testosterone levels in the castrated *Efnb3* KO males. Therefore, for the *ADVANCE* patients with *EFNB3* mutations, they might have lost the protective effect of testosterone. Indeed, some reports indicate that hypogonadism in men, whether evoked by ageing, diabetes, chemical castration or other unidentified factors, correlates with hypertension^53^,^54^,^55^,^56^,^57^,^58^,^59^. It is possible that some of these hypogonadic hypertension patients suffer from *EFNB3* mutations, and the concerted effect of lower testosterone levels and *EFNB3* mutation renders them prone to hypertension, as is the case in castrated *Efnb3* KO mice. 2) Another contributing factor could be that these T2D males are generally overweight or obese. Adipose tissues are rich in aromatase, a rate-limiting enzyme converting testosterone to estrogen^60^. Therefore, obese males tend to have relatively higher estrogen levels, which in the absence of EFNB3, could increase BP, as we have demonstrated in our *Efnb3* KO mouse model^29^. Hence, the *ADVANCE* cohort provides us with a unique situation to reveal the association of the *EFNB3* SNPs with hypertension in these male T2D patients. 3) In this genetic study, we demonstrated that 2 SNPs in the *EFNB3* genes were associated with hypertension in males, but the association could be either positive or negative. If the association is negative in male humans, it is not incompatible with our findings that male KO mice show no increased BP.

In the discussion of the two previous paragraphs regarding the discrepant results from the *Efnb3* KO mouse model versus those from the current *ADVANCE* study regarding the sex differences, we assumed that the mutation in the *EFNB3* gene in this study is a loss-of-function one. However, there is no evidence that this is the case. The responsible mutations in LD with the 2 significant SNPs could actually result in alteration-of-function or gain-of-function. If that is the case, there would be concordance between our findings that *EFNB3* deletion in mice causes increased BP in females, while in humans, the 2 *EFNB3* SNPs are associated with hypertension in males, as alteration-of-function and gain-of-function mutations could have quite different consequences from loss-of-function mutations. This possibility discussed in this paragraph would override the explanations in the previous two paragraphs.

In summary, in this study, we discovered a significant association of 2 SNPs in the *EFNB3* gene with hypertension in a human genetic study. This has opened a new area of investigation into the pathogenesis, diagnosis and personalized therapy of hypertension.

## Additional Information

**How to cite this article:** Tremblay, J. *et al*. Evidence from single nucleotide polymorphism analyses of ADVANCE study demonstrates *EFNB3* as a hypertension risk gene. *Sci. Rep.* **7**, 44114; doi: 10.1038/srep44114 (2017).

**Publisher's note:** Springer Nature remains neutral with regard to jurisdictional claims in published maps and institutional affiliations.

## Figures and Tables

**Table 1:** Numbers and percentages of hypertension cases and controls based on 140/90 mmHg or 160/100 mmHg cut-offs.

Cases		number	% total	mean SBP (mmHg)	SD	mean DSP (mmHg)	SD
**Males**							
140/90 mmHg cut-off	Actively treated	1264	**70.5**	149.5	20.7	82.9	10.7
	>140/90 mmHg; not treated	530	29.5	156.0	14.6	85.2	9.3
	Total	1794					
160/100 mmHg cut-off	Actively treated so cannot be used	1264	**87.2**	149.5	20.7	82.9	10.7
	>160/100 mmHg; not treated	185	12.8	171.7	12.0	90.2	8.9
	Total	1449					
Controls							
140/90 mmHg cut-off	≤140/90 mmHg but ≥130/80 mmHg	425	**81.7**	126.6	9.2	74.8	7.3
130/80 mmHg cut-off	≤130/80 mmHg	95	18.3	119.2	7.7	70.3	5.9
	Total	520					
**Females**							
140/90 mmHg cut-off	Actively treated	801	**78.8**	150.1	22.7	81.6	11.2
	>140/90 mmHg; not treated	215	21.2	156.3	14.5	83.7	9.1
	Total	1016					
160/100 mmHg cut-off	Actively treated so cannot be used	801	**92.4**	150.1	22.7	81.6	11.2
	>160/100 mmHg; not treated	66	7.6	176.4	16.4	90.5	11.0
	Total	867					
Controls							
140/90 mmHg cut-off	<140/90 mHg but ≥130/80 mmHg	213	**69.2**	126.8	9.7	73.4	7.4
130/80 mmHg cut-off	<130/80	95	30.8	118.9	8.1	69.5	5.5
	Total	308					

The numbers and percentages of cases and controls recruited into the study are provided based on whether they were actively treated, or based on BP measurements at the entry of the study. This information is calculated on 2 putative different BP cut-offs, *i.e*., 1) >140 or >90 mmHg for cases, and <140 and <90 mmHg for controls; 2) >160 or >100 mmHg for cases and <130 and <80 mmHg for controls. Means and SD of SBP (systolic blood pressure) and DBP (diastolic blood pressure) are also listed. For those cases actively treated for hypertension, since we do not have their real BP reading in the absence of medication, and they cannot be used in the in the high cut-off scenario, they are labels as “Actively treated so cannot be used” in the 160/100 mmHg cut-off group.

**Table 2:** Association analysis for 39 SNPs covering the region of the EFNB3 gene with an additional 10 kb 5′ and 3′ of the gene for the combined male and female sample, the male-only sample and the female-only sample.

SNP ID	Position Chromosome 17 (Build 37/hg19)	Coding Allele	Alternate Allele	Minor Allele	^a^MAF	All			Males			Females		
						^*b*^*p*-value Association	β coefficient logistic Regresssion	Odds Ratio for Coding Allele	^*b*^*p*-value Association	β coefficient logistic Regresssion	Odds Ratio for Coding Allele	^*b*^*p*-value Association	β coefficient logistic Regresssion	Odds Ratio for Coding Allele
rs12939910	7599260	G	C	G	0.083	0.12477	−0.187	0.812	0.02836	−0.328785	0.734155	0.87478	0.033	1.001
rs12938947	7599665	A	G	A	0.082	0.10526	−0.199	0.805	0.02693	−0.334713	0.729201	0.96505	0.009	0.989
rs12950295	7599851	C	T	C	0.175	0.07954	−0.166	0.854	0.16536	−0.160257	0.854119	0.20450	−0.209	0.840
rs12939581	7599859	A	G	A	0.083	0.04861	−0.241	0.778	0.03350	−0.316053	0.726025	0.48343	−0.152	0.900
rs11659090	7600926	G	A	G	0.175	0.21216	−0.117	0.877	0.25533	−0.130554	0.867979	0.47988	−0.114	0.878
rs12941981	7608462	A	C	A	0.092	0.13720	−0.180	0.831	0.04492	−0.296001	0.764927	0.95660	0.011	0.988
^**c**^**rs3744263**	7613708	C	T	T	0.320	**0.00034**	**0.253**	**1.317**	**0.00195**	**0.267526**	**1.36367**	0.04734	0.242	1.242
rs3744262	7613765	A	G	G	0.365	0.00572	0.194	1.243	0.01931	0.201133	1.2752	0.10881	0.194	1.191
^**c**^**rs7141**	7614601	A	G	G	0.304	**0.00026**	**0.268**	**1.319**	**0.00148**	**0.28582**	**1.36416**	0.03779	0.261	1.249
rs56371490	7615143	T	C	C	0.362	0.01127	0.179	1.222	0.03116	0.187369	1.2527	0.13850	0.180	1.172
rs9907011	7615286	T	C	T	0.158	0.01191	−0.234	0.792	0.02361	−0.261224	0.771113	0.20641	−0.198	0.817
rs3948593	7615364	C	T	T	0.146	0.03067	0.208	1.238	0.07727	0.205784	1.2701	0.14138	0.250	1.169
rs62062589	7615476	C	G	G	0.352	0.01011	0.182	1.231	0.03060	0.187404	1.26038	0.11540	0.192	1.185
rs9906502	7615745	A	G	A	0.167	0.00860	−0.241	0.789	0.01752	−0.269905	0.766975	0.18733	−0.204	0.816
rs67022015	7617165	C	T	C	0.098	0.11132	−0.181	0.820	0.06851	−0.254179	0.769694	0.70236	−0.075	0.922
rs12943024	7617209	T	C	T	0.096	0.08925	−0.195	0.812	0.05425	−0.270334	0.760844	0.67087	−0.084	0.918
rs62062590	7617378	A	G	A	0.317	0.01447	0.177	1.271	0.00985	0.222777	1.34282	0.40259	0.109	1.154
rs35547626	7617558	C	T	C	0.320	0.03598	0.153	1.242	0.03252	0.186881	1.29198	0.43058	0.103	1.160
rs62062591	7617572	A	C	A	0,317	0.02770	0.160	1.249	0.02400	0.196057	1.30176	0.41011	0.108	1.161
rs11867748	7617662	A	C	A	0.098	0.10942	−0.182	0.819	0.06586	−0.257191	0.766705	0.70787	−0.073	0.924
rs11870307	7617787	G	A	A	0.202	0.00298	0.257	1.269	0.01832	0.24949	1.29048	0.05062	0.290	1.241
rs60370790	7617955	A	G	A	0.361	0.26425	0.079	1.038	0.57855	0.0481182	1.027	0.27390	0.135	1.058
rs62062593	7618026	C	T	C	0.309	0.02570	0.162	1.257	0.01802	0.204916	1.32361	0.46836	0.095	1.147
rs11651917	7618604	A	G	A	0.301	0.03249	0.156	1.253	0.01914	0.203731	1.32687	0.55684	0.077	1.131
rs34873228	7618737	T	C	T	0.098	0.09977	−0.188	0.818	0.06709	−0.256201	0.767848	0.65493	−0.088	0.921
rs1123547	7619861	C	T	C	0.363	0.25014	0.081	1.041	0.56717	0.0492604	1.02779	0.25625	0.138	1.067
rs7208469	7619871	A	G	A	0.303	0.02661	0.161	1.259	0.01474	0.211384	1.3393	0.56536	0.075	1.127
rs4246412	7620595	T	G	T	0.363	0.24388	0.082	1.043	0.58159	0.0472188	1.02484	0.22775	0.146	1.078
rs4968206	7620702	G	T	G	0.477	0.85398	0.012	0.963	0.72738	−0.0287709	0.936648	0.44371	0.090	1.013
rs4968207	7620907	G	C	G	0.355	0.36763	0.064	1.025	0.70825	0.0324189	1.01287	0.31750	0.122	1.049
rs3803802	7621464	A	C	A	0.465	0.76738	0.020	0.971	0.73459	−0.027465	0.936317	0.37628	0.101	1.038
rs7359524	7621528	A	G	A	0.294	0.02767	0.162	1.257	0.01413	0.215663	1.34071	0.60819	0.068	1.121
rs58049067	7621561	C	T	C	0.097	0.13675	−0.172	0.830	0.07388	−0.254645	0.771343	0.81378	−0.047	0.950
rs1544724	7621777	G	T	G	0.464	0.78804	0.018	0.970	0.73992	−0.0269205	0.937495	0.40859	0.095	1.033
rs1544725	7621877	A	C	A	0.087	0.10294	−0.197	0.800	0.07950	−0.260991	0.756051	0.56762	−0.119	0.896
rs3744260	7621943	A	G	A	0.366	0.22459	0.085	1.043	0.45851	0.06344	1.03401	0.30109	0.125	1.061
rs3744259	7622209	A	G	A	0.363	0.25200	0.081	1.041	0.58521	0.046926	1.02387	0.23738	0.144	1.075
rs3744258	7623394	G	A	G	0.368	0.26200	0.080	1.035	0.50276	0.0585537	1.02531	0.33854	0.118	1.052
rs307629	7624326	C	T	C	0.100	0.56032	−0.071	0.908	0.26781	−0.165516	0.846448	0.72667	0.072	1.036

^a^MAF = minor allele frequency.

^b^Bonferroni corrected p-value for 3 categories (all, male, female) X 8 tag SNPs = 8 LD blocks = 24 independent tests = 0.05/21 = 0.002381; *Bonferroni* significant *p*-values are in bold.

^c^rs: reference SNP cluster ID. rs7141 and rs3744263: the two *Bonferroni* significant SNPs, are in LD at *r*^*2*^ = 0.896 in Utah CEU population (SNAP version 2.2 Broad Institute, 1000 genomes data).

**Table 3:** Odds ratios and 95% confidence intervals for odds ratios for SNPs rs7141 and rs3744263 for combined male and female samples, for males-only samples and for females-only samples.

	Combined Males and Females			Males Only				Females Only		
	Cases	Controls	Odds Ratio for Allele A or C ^a^(95% CI)	Cases	Controls	Odds Ratio for Allele A or C ^a^(95% CI)		Cases	Controls	Odds Ratio for Allele A or C ^a^(95% CI)
SNP *rs7141* (imputed)										
A	N	3972	824	1.319	2562	550	1.364	1411	275	1.249
	Frequency	0.707	0.646	(1.159–1.498)	0.714	0.647	(1.163–1.600)	0.694	0.645	(1.000–1.554)
G	N	1646	450		1026	300		621	151	
	Frequency	0.293	0.354		0.286	0.353		0.306	0.355	
SNP *rs3744263* (genotyped)										
C	N	3883	802	1.317	2506	535	1.364	1378	268	1.242
	Frequency	0.691	0.630	(1.159–1.498)	0.698	0.629	(1.166–1.595)	0.678	0.629	(0.999–1.544)
T	N	1735	472		1082	315		654	158	
	Frequency	0.309	0.370		0.302	0.371		0.322	0.371	

^a^95% CI = 95% confidence interval of odds ratio.

**Table 4:** Tests for independence of sexes with respect to allele frequencies for SNPs rs7141 and rs3744263.

		Cases			Control		
		Males	Females	χ^2^ test of independence (*p*-value)	Males	Females	χ^2^ test of independence (*p*-value)
SNP *rs7141* (imputed)							
A	N	2562	1411	2.420	550	275	
	Frequency	0.714	0.694	(0.120)	0.647	0.645	0.003
G	N	1026	621		300	151	(0.957)
	Frequency	0.286	0.306		0.353	0.355	
SNP *rs3744263* (genotyped)							
C	N	2506	1378	2.502	535	268	0.0001
	Frequency	0.698	0.678	(0.114)	0.629	0.629	(0.992)
T	N	1082	654		315	158	
	Frequency	0.302	0.322		0.371	0.371
